# From Bed to Bench and Back: TNF-α, IL-23/IL-17A, and JAK-Dependent Inflammation in the Pathogenesis of Psoriatic Synovitis

**DOI:** 10.3389/fphar.2021.672515

**Published:** 2021-06-15

**Authors:** Ettore Silvagni, Sonia Missiroli, Mariasole Perrone, Simone Patergnani, Caterina Boncompagni, Alessandra Bortoluzzi, Marcello Govoni, Carlotta Giorgi, Stefano Alivernini, Paolo Pinton, Carlo Alberto Scirè

**Affiliations:** ^1^ Rheumatology Unit, Department of Medical Sciences, Università degli Studi di Ferrara and Azienda Ospedaliero-Universitaria S. Anna, Cona, Italy; ^2^ Department of Medical Sciences, Section of Experimental Medicine, Laboratory for Technologies of Advanced Therapies, University of Ferrara, Ferrara, Italy; ^3^ Rheumatology Unit, Fondazione Policlinico Universitario A. Gemelli IRCCS, Rome, Italy; ^4^ Epidemiology Research Unit, Italian Society for Rheumatology, Milan, Italy

**Keywords:** psoriatic arthritis, TNF-alfa, IL-23/IL-17 axis, JAK/STAT pathway, synovial histopathology, synovial biopsy, targeted therapies, personalized medicine

## Abstract

Psoriatic arthritis (PsA) is a chronic inflammatory immune-mediated disease with a burdensome impact on quality of life and substantial healthcare costs. To date, pharmacological interventions with different mechanisms of action, including conventional synthetic (cs), biological (b), and targeted synthetic (ts) disease-modifying antirheumatic drugs (DMARDs), have been proven efficacious, despite a relevant proportion of failures. The current approach in clinical practice and research is typically “predictive”: the expected response is based on stratification according to clinical, imaging, and laboratory data, with a “heuristic” approach based on “trial and error”. Several available therapeutic options target the TNF-α pathway, while others are directed against the IL-23/IL-17A axis. Janus kinase inhibitors (JAKis), instead, simultaneously block different pathways, endowing these drugs with a potentially “broad-spectrum” mechanism of action. It is not clear, however, whether targeting a specific pathway (e.g., TNF-α or the IL-23/IL-17 axis) could result in discordant effects over other approaches. In particular, in the case of “refractory to a treatment” patients, other pathways might be hyperactivated, with opposing, synergistic, or redundant biological significance. On the contrary, refractory states could be purely resistant to treatment as a whole. Since chronic synovitis is one of the primary targets of inflammation in PsA, synovial biomarkers could be useful in depicting specific biological characteristics of the inflammatory burden at the single-patient level, and despite not yet being implemented in clinical practice, these biomarkers might help in selecting the proper treatment. In this narrative review, we will provide an up-to-date overview of the knowledge in the field of psoriatic synovitis regarding studies investigating the relationships among different activated proinflammatory processes suitable for targeting by different available drugs. The final objective is to clarify the state of the art in the field of personalized medicine for psoriatic disease, aiming at moving beyond the current treatment schedules toward a patient-centered approach.

## Introduction

Psoriatic arthritis (PsA) is a chronic systemic immune-mediated inflammatory disease belonging to the spondyloarthritis (SpA) spectrum. PsA occurs at frequencies ranging from 6% to 42% of patients with skin psoriasis, according to different studies (Scher et al., 2019; Zabotti et al., 2020), or affects family members of psoriatic patients (Gladman et al., 2005). Skin disease is considered the main risk factor for PsA development, and although the occurrence of joint disease is not predictable, an incidence risk of approximately 20% is approximated after more than 30 years of skin psoriasis, with higher rates in the context of nail, scalp, or inverse psoriasis; this risk is also affected by the severity of the cutaneous manifestations. In the context of PsA, involvement of the joints, entheses, and skin is challenging for clinicians, and dactylitis, nail dystrophy, uveitis, and spine manifestations represent clinical endotypes susceptible to different management approaches. Progressive damage accrual, along with inflammatory manifestations of the disease, is highly disabling for patients, with impacts on quality of life and healthcare costs (Singh and Strand, 2009). Comorbidities associated with repercussions related to cardiovascular risk, such as obesity and metabolic syndrome, are intrinsic parts of psoriatic “disease”.

This clinical heterogeneity is reflected in complex pathophysiology, knowledge of which is crucial to hypothesizing a therapeutic approach targeting the ongoing pathological process (Veale and Fearon, 2018; Scher et al., 2019; Russell et al., 2021). Infiltration of both innate and adaptive immune cells in different target organs and tissues results in significant production of different proinflammatory cytokines, including tumor necrosis factor-α (TNF-α), interleukin (IL)-1β, IL-6, IL-22, IL-23, IL-17A, and IL-18, inducing further inflammatory mediators release and damage progression. New evidence in PsA pathogenesis has provided further insights into the molecular pathways involved in either cutaneous or articular manifestations of the disease, and genetic, epigenetic, environmental, cellular, and molecular aspects have been clarified, driving the development of different targeted therapies (Jadon et al., 2020). From a clinical point of view, drugs targeting different molecules primarily involved in chronic inflammation, such as TNF-α, the IL-23/IL-17A axis, or Janus kinases/signal transducers and activators of transcription (JAK/STATs), are now available (Gossec et al., 2016, 2020; Singh et al., 2018). This multiplicity of treatment options poses relevant questions about how to best interfere with different proinflammatory processes in individual PsA patients because clinicians lack reliable tools to select the best therapeutic pathway to target to optimize clinical response. A priori, tissue-specific biomarkers are the most promising candidates to stratify patients based on the actual ongoing pathogenic process and are suitable for targeting by pharmacological treatments, as demonstrated by some attempts in other chronic inflammatory joint diseases, such as rheumatoid arthritis (RA) (Humby et al., 2021). Despite important discoveries in this field, there are still great obstacles to the goal of “personalized arthritis medicine’ in the context of PsA (Jadon et al., 2020).

The main aim of this narrative review is to provide an up-to-date overview of the knowledge in the field of psoriatic synovitis, focusing specifically on studies investigating the relationships among activated proinflammatory immune pathways, following hyperactivation of TNF-α, the IL-23/IL-17A axis, or JAK/STAT-dependent inflammation. Moreover, we will summarize the most robust and innovative evidence on the synovial membrane as a biomarker of response to treatment in PsA patients.

### Targeted Therapies for Psoriatic arthritis

Bedside data on the efficacy of treatments for PsA come from randomized controlled trials (RCTs) and observational studies, as well as systematic literature reviews (SLRs) (Kerschbaumer et al., 2020) informing current clinical practice guidelines (Coates et al., 2016; Gossec et al., 2016, Gossec et al., 2020; Singh et al., 2018). First-line pharmacological treatment strategies include nonsteroidal anti-inflammatory drugs (NSAIDs) and/or local injection of glucocorticoids (GCs). Conventional synthetic (cs) disease-modifying antirheumatic drugs (DMARDs) (e.g., methotrexate (MTX)) are selected in the case of elective peripheral joint involvement, while PsA patients refractory to csDMARDs should be treated with biological (b) DMARDs or oral targeted synthetic (ts) DMARDs. Current treatment guidelines suggest the use of JAK inhibitors (JAKis) in the case of bDMARD treatment failure or when other biologics are contraindicated. Since the amount of data on JAKis adoption in PsA will increase in the coming years, the positioning of these drugs might be revised when treatment recommendations are updated. This stepwise approach is generally accepted worldwide. Currently available bDMARDs for the management of PsA, which were designed based on the growing knowledge of disease pathogenesis, include 5 different TNF inhibitors (TNFis) (infliximab (IFX), etanercept (ETA), adalimumab (ADA), certolizumab pegol (CTZ), golimumab (GOL)), and their available biosimilars, the anti-IL-12/IL-23 p40 common subunit antibody ustekinumab, the anti-IL-17A antibodies secukinumab (SEC) and ixekizumab (IXE), and the selective T-cell co-stimulation modulator abatacept. Additionally, apremilast, tofacitinib, and upadacitinib are the only oral tsDMARDs available for PsA approved by the Food and Drug Administration (FDA) and European Medicine Association (EMA). The first inhibits phosphodiesterase-4 (PDE4). Tofacitinib blocks JAK1 and JAK3, with a functional effect on JAK2 (Mease et al., 2017a; Gladman et al., 2017), while upadacitinib is a selective JAK1 inhibitor (Mease et al., 2021b). Moreover, other drugs for the systemic management of PsA are in different phases of development. The anti-IL-23 biologics risankizumab (Clinical trial registration, KEEPsAKE 1, 2021; Clinical trial registration, KEEPsAKE 2, 2021), tildrakizumab (Clinical trial registration, INSPIRE 1, 2021; Clinical trial registration, INSPIRE 2, 2021), and guselkumab (Mease et al., 2020a; Deodhar et al., 2020) and the anti-IL17 receptor (IL-17R) antibody brodalumab (Mease et al., 2021a), which is already approved for psoriasis management, are under investigation in PsA, and the bispecific immunoglobulin bimekizumab, which targets IL-17A and IL-17F, is also being studied (Ritchlin et al., 2020a). Among tsDMARDs, filgotinib is promising (Clinical trial registration, PENGUIN 1, 2021; Clinical trial registration, PENGUIN 2, 2021). Table 1 summarizes the drugs for the management of PsA that are currently approved or in different phases of development.

**TABLE 1:** b/tsDMARDs that have been approved or are in different phases of development for the systemic management of PsA.

bDMARDs	Mechanism of action	EMA/FDA approval or phase of development
Etanercept	TNFi	RA; PsA; PsO; JIA; AS/nrAS
Adalimumab	TNFi	RA; PsA; PsO; JIA; AS/nrAS; SH; CD/UC; chronic uveitis
Infliximab	TNFi	RA; PsA; PsO; AS/nrAS; CD/UC
Golimumab	TNFi	RA; PsA; JIA; AS/nrAS; UC
Certolizumab pegol	TNFi	RA; PsA; PsO; AS/nrAS
Ustekinumab	p40 common subunit (IL-12 and IL-23) inhibitor	PsA; PsO; CD/UC
Secukinumab	IL-17A inhibitor	PsA; PsO; AS/nrAS
Ixekizumab	IL-17A inhibitor	PsA; PsO; AS/nrAS
Abatacept	CD80/CD86-mediated	RA; PsA
	Co-stimulation inhibitor	
Risankizumab	p19 subunit (IL-23) inhibitor	PsO
		*PsA (phase II trial completed and phase III trials ongoing)*
Tildrakizumab	p19 subunit (IL-23) inhibitor	PsO
		*PsA (phase II trial completed and phase III trials ongoing)*
Guselkumab	p19 subunit (IL-23) inhibitor	PsO
		*PsA (phase II–III trials completed)*
Brodalumab	IL-17RA inhibitor	PsO
		*PsA (phase III trials completed)*
Bimekizumab	IL-17A and IL-17F bispecific antibody	*PsO (phase IIb trial completed and phase III trials ongoing)*
		*PsA (phase IIb trial completed and phase III trials ongoing)*
tsDMARDs	Mechanism of action	EMA/FDA approval or phase of development
Apremilast	PDE4 inhibitor	PsA; PsO; BD
Tofacitinib	JAK1/3 inhibitor	RA; PsA; UC
Upadacitinib	JAK1 inhibitor	RA; PsA
Filgotinib	JAK1 inhibitor	RA; *PsA (phase II completed and phase III trials active—not recruiting)*

b/tsDMARDs, biological/targeted synthetic disease-modifying antirheumatic drugs; PsA, psoriatic arthritis; EMA, European Medicines Agency; FDA, U.S. Food and Drug Administration; TNFi, tumor necrosis factor-alpha inhibitor; RA, rheumatoid arthritis; PsO, psoriasis; JIA, juvenile idiopathic arthritis; AS/nrAS, ankylosing spondylitis/nonradiographic axial spondylarthritis; SH, suppurative hidradenitis; CD, Crohn’s disease; UC, ulcerative colitis; IL, interleukin; IL-17RA, IL-17 receptor A; PDE4, phosphodiesterase-4; BD, Behcet’s disease; JAK, Janus kinase.

Among the high number of drugs registered for the management of PsA in recent years, the majority of available therapeutic options act against the TNF-α pathway, while others are directed against the IL-23/IL-17A axis, and JAKis virtually encompass the intersections of a number of pathways based on their potential “broad-spectrum” mechanism of action, blocking different type I and II cytokines (e.g., IL-6, IL-23, IL-22, and interferons (IFNs)). This wide availability of drugs enables interference with the most important cytokines and nodes involved in disease pathogenesis, with the clinical aim of reducing signs and symptoms of the disease and preventing joint/bone damage and disability accrual. From a pathogenetic point of view, instead, the possibility of interfering with single or multiple crossroads directly involved in disease susceptibility and synergism could reduce inflammation in its entirety at the site of the disease (i.e., the skin, entheses, and synovium), decelerating the progression to more advanced stages of illness.

In regard to bedside application, the choice of the preferred bDMARD as a first-line biological treatment in patients with peripheral arthritis and selection of the correct strategy after failure of the first bDMARD (historically a TNFi) are aspects of interest, despite available evidence supporting clinical decisions being scant (Silvagni et al., 2019; Chimenti et al., 2020). Indeed, after almost 20 years of TNFi availability in the field of PsA, the capability to treat PsA with this class of drugs in the clinic has been reinforced by long-term efficacy and safety data (Fagerli et al., 2018; Haugeberg et al., 2018). TNFis are usually considered among first-line biological treatment strategies in different clinical settings, while experience with recently developed anti-IL-17A agents is obviously lower. However, similar efficacy rates have been shown between TNFis and non-TNFis in RCTs, with higher responses in first-line treatment strategies (McInnes et al., 2013; Mease et al., 2017b) than in second-line options (Ritchlin et al., 2014; McInnes et al., 2015; Mease et al., 2015; Nash et al., 2017). Moreover, the recent European League Against Rheumatisms (EULAR) recommendations suggested preferring an anti-IL-17A or anti-IL-12/23 agent in cases with relevant skin involvement (Gossec et al., 2020), and this remains, at present, the only acknowledgment of personalized systemic treatment in this context. However, in the 2018 American College of Rheumatology (ACR) guidelines, TNFis are conditionally suggested as the first-line treatment strategy over anti-IL-17A and anti-IL-12/23 antibodies (Singh et al., 2018) on the basis of the more robust amount of clinical data.

Information regarding the comparative effectiveness of drugs with different modes of action in PsA is steadily increasing. Indirect evidence from RCTs and observational studies cautiously suggests a higher efficacy for IL-17A inhibition at the cutaneous level, with respect to joint involvement, while the use of TNFis has produced more comparable rates of response between the skin and joints (Boutet et al., 2018). This was confirmed by network meta-analyses. TNFis demonstrated substantially higher ACR responses (i.e., articular symptoms) than other biologics and tsDMARDs, although the differences were numerically low (Gladman et al., 2020; Ruyssen-Witrand et al., 2020), and the effect of prior exposure to bDMARDs did not result in higher efficacy for other drugs with different mechanisms of action. On the contrary, both TNFis (except for etanercept) and anti-IL17A agents produced consistent cutaneous responses (i.e., Psoriasis Area Severity Index (PASI) response) compared to placebo. Recently, head-to-head RCTs directly comparing different active treatment strategies have provided relevant practical information. In the EXCEED trial (McInnes et al., 2020), 853 active bDMARD-naïve PsA patients were randomly assigned to the anti-IL-17A SEC (300 mg monthly) or the TNFi ADA, with the primary objective of demonstrating the superiority of SEC over ADA at 52 weeks (ACR20 response). The primary endpoint was not reached; however, SEC demonstrated an efficacy profile similar to that of ADA (odds ratio [OR]: 1.30, 95% confidence interval [95% CI]: 0.98–1.72) with no new safety signals with respect to registration RCTs. In the SPIRIT-H2H trial (Mease et al., 2020b; Smolen et al., 2020), the primary objective was the simultaneous achievement of joint and skin responses (ACR50 and PASI100) with IXE compared to ADA. IXE was superior to ADA in terms of the combined skin and joint primary endpoint (*p* = 0.036 at 24 weeks, *p <* 0.001 at 52 weeks) and non-inferior to ADA in ACR responses. Additional RCTs compared TNFis with non-TNFis, and although these trials were not designed to primarily evaluate the comparison, numerical differences in terms of efficacy endpoints, at least for the peripheral joints involvement, were not clinically meaningful (Mease et al., 2017b; Coates et al., 2017). Table 2 highlights the differences in clinical responses between TNFis and non-TNFis in phase III RCTs in PsA comparing different treatment arms.

**TABLE 2:** Main differences in clinical response between TNFis and non-TNF b/tsDMARDs in PsA phase III RCTs directly comparing the treatment arms.

Study	Treatment arms	Population	Primary objective	Results
EXCEED trial, McInnes et al. (2020)	SEC 300 mg or ADA 40 mg	Active bDMARDs-naïve PsA	Superiority of SEC vs ADA for ACR20 at 52 weeks	Primary objective not met (SEC was noninferior to ADA, SEC 67%; ADA 59%, *p* = 0.0239).
SPIRIT-H2H trial, Mease et al. (2020b); Smolen et al. (2020)	IXE 160/80 mg or ADA 40 mg	Active bDMARDs-naïve PsA	Superiority of IXE vs ADA for simultaneous achievement of ACR50 and PASI100 at 24 weeks	IXE was superior to ADA (IXE 36%; ADA 28%, *p* = 0.036); results were maintained at 52 weeks (IXE 39%; ADA 26%, *p <* 0.001).
Enthesial CLearance In PSoriatic Arthritis (ECLIPSA), Araujo et al. (2019)	UST 45/90 mg or TNFi (open label)	Active bDMARDs-naïve PsA with active enthesitis	SPARCC = 0 at 24 weeks	UST was superior to TNFis (UST 74%; TNFis 42%, *p* = 0.018) but not at the joint levels (SJC + TJC = 0: UST 41%; TNFis 34%).
SPIRIT-P1 study, Mease et al. (2017b); Coates et al. (2017)	IXE 80 mg every 4 weeks, IXE 80 mg every 2 weeks, ADA 40 mg, or placebo	Active csDMARDs-IR PsA	Superiority of IXE vs placebo for ACR20 at 24 weeks	IXE was superior to placebo, similar effect compared to ADA (IXE 4 W 58%; IXE 2 W 62%; placebo 30%, *p <* 0.001; ADA 57%).
Oral Psoriatic Arthritis Trial (OPAL) Broaden, Mease et al. (2017a)	Tofacitinib 5 mg BID or 10 mg BID, ADA 40 mg, or placebo	Active csDMARDs-IR PsA	Superiority of tofacitinib vs placebo for ACR20 at 12 weeks	Tofacitinib was superior to placebo, similar effect compared to ADA (Tofa 5 mg BID 50%; Tofa 10 mg BID 61%; placebo 33%, *p <* 0.001; ADA 52%).

TNFis, TNF inhibitors; b/tsDMARDs, biological/targeted synthetic disease-modifying antirheumatic drugs; PsA, psoriatic arthritis; RCTs, randomized controlled trials; SEC, secukinumab; ADA, adalimumab; ACR, American College of Rheumatology; ACR20, ACR response 20%; IXE, ixekizumab; PASI, Psoriasis Area Severity Index; UST, ustekinumab; W, week; SPARCC, Spondyloarthritis Research Consortium of Canada; csDMARDs-IR, conventional synthetic DMARDs insufficient responders; BID, bis in die.

In line with these results, the decision on the biologic to adopt in each PsA patient is substantially empirical, and guidelines are not restrictive in this sense, with even more elusive data on the sequencing of therapies. EULAR recommendations (Gossec et al., 2020) suggest a preferred option for an anti-IL-17A or anti-IL-12/23 agent over TNFis and JAKis in cases with severe skin involvement, while the 2015 Group for Research and Assessment of Psoriasis and Psoriatic Arthritis (GRAPPA) guidelines tried to enlist specific treatments for individual clinical domains (e.g., axial, enthesis, dactylitis, and peripheral joint) and in cases with selected comorbidities (Ogdie et al., 2020). However, individual treatment decisions remain based on “heuristic” approaches, being not tailored to the biological features of the disease. Based on these issues, this approach exposes patients to possible primary inefficacy, unexpected side effects or several failed biological treatments before achieving clinical amelioration. As almost 40% of patients do not appropriately respond to their first-line biological treatment (Ritchlin et al., 2020b), the search for predictive biomarkers able to depict treatment response a priori is one the major unmet needs in the field, and addressing this need includes a global reconsideration of RCT development, aiming to tailor treatment decisions at the “single-patient” level (Miyagawa et al., 2018; Leijten et al., 2019; Pitzalis et al., 2020).

### Treatment Response Biomarkers in Psoriatic Arthritis

As underlined above, determining biomarkers related to early diagnosis, damage, prognosis, and treatment response is one of the major unmet needs in the field of PsA; as such, it is included in the research agendas of the most relevant international treatment guidelines (Ogdie et al., 2020). Biomarkers are currently defined as measurable indicators of disease status. Despite the growing number of studies aimed at identifying diagnostic, prognostic, and treatment selection biomarkers (Generali et al., 2016; Mahendran and Chandran, 2018; Mahmood et al., 2018), no validated biomarkers are yet available for clinical use in PsA (Scher et al., 2019). Special interest lies in identifying peripheral blood, synovial fluid (SF), and synovial membrane biomarkers of response to drugs with different mechanisms of action (Jadon et al., 2020).

#### Genetic Biomarkers

Genetic biomarkers to predict clinical response, mostly to TNFis, have been investigated in both psoriasis and PsA (O’Rielly et al., 2019). Early reports require confirmation in defined clinical subsets, with homogenization of inclusion criteria in clinical presentation, course of the disease, and the genotyping and molecular expression of specific cells and tissues. Polymorphisms in the TNFAIP3 (Ovejero-Benito et al., 2019), TNF-α 308A, IL-6 174 (Fabris et al., 2016), and TNF489A (Murdaca et al., 2014) alleles were related to the clinical efficacy of different TNFis in observational studies. Genetic and epigenetic modifications have also been exploited to highlight the treatment response to other bDMARDs, such as ustekinumab, but the evidence is mostly available in psoriasis rather than PsA (Ovejero-Benito et al., 2018; Dand et al., 2019). Since the amount of research data will increase as the availability of new b/tsDMARDs increases, the amount of genetic biomarker data will rise accordingly. However, PsA is a multifactorial disease, and genetic predisposition accounts for only a portion of the pathogenic process, with environmental factors significantly influencing the course of this disease (Veale and Fearon, 2018). Thus, it is not surprising that genetic biomarkers have not yet entered clinical practice.

#### Serum Biomarkers

Since serum biomarkers are the most easily accessible measures, a relatively high number of studies have focused on serum levels of different proinflammatory molecules, clearly demonstrating increased levels of IL-17A, IL-23, IL-6, IL-1β, IL-21, transforming growth factor (TGF)-β, TNF-α, and interferon-gamma (IFN-γ) in the serum and SF of patients with SpA, including PsA, compared to those of controls (Londono et al., 2012; Raychaudhuri and Raychaudhuri, 2017). Among biomarkers of response to treatment, baseline C-reactive protein (CRP), IL-6 (Muramatsu et al., 2017), matrix metalloproteinase 3 (MMP-3) (Chandran et al., 2013), low-molecular-mass hyaluronan (Hellman et al., 2019), and C3 levels (Chimenti et al., 2012) were found to be predictive of TNFi therapy response in prospective studies (Gratacós et al., 2007; Kristensen et al., 2008; Scrivo et al., 2020). Lower IL-6 levels were associated with clinical response to ustekinumab (Muramatsu et al., 2017). Longitudinal decreases in the plasma concentrations of IL-6, vascular endothelial growth factor (VEGF), MMP3, and chitinase-3 like-1 (YKL-40) (Pedersen et al., 2010) and increases in serum cartilage oligomeric matrix protein (COMP) (Chandran et al., 2013) levels were linked to clinical response to TNFis. Moreover, from a panel of 92 serum proteins, pyridinoline, adiponectin, prostatic acid phosphate (PAP), and factor VII were identified as predictors of response to golimumab in a prospective observational study (Wagner et al., 2013). However, the serum concentrations of metabolites are influenced by several factors, and although PsA is a systemic condition, none of these biomarkers have been validated in clinical trials. Therefore, their roles are mostly mechanistic rather than decisional.

#### Peripheral Blood Cellular Biomarkers

Within the cellular compartment, several studies have demonstrated elevated frequencies of IL-17-positive T-cells in patients with PsA (Leipe et al., 2010; Dolcino et al., 2015), with even higher numbers in the SF (Raychaudhuri et al., 2012; Menon et al., 2014). Peripheral T-cell phenotyping was exploited in one of the first attempts to apply a precision medicine approach in PsA. Miyagawa et al. (2018) directly compared, across a proof-of-concepts open-label study, two different treatment strategies in a population of patients with active PsA and an insufficient response to MTX (26 patients in a strategic treatment group versus 38 in a standard administration group following EULAR recommendations). Before starting therapy, FACS analysis of peripheral blood lymphocytes was performed to phenotypically characterize circulating T cells. Patients with a higher T helper 1 (Th1) cell status received the anti-IL-12/IL-23 antibody ustekinumab, while those with a higher Th17 cell level were treated with the IL-17A blocker SEC. A TNFi or SEC was given if the peripheral blood T-cell population was enriched in both the Th1 and Th17 clusters, while only the TNFi was administered when both were downregulated. This tailored approach with specific interventions based on distinct T-cell phenotypes and presumed activated proinflammatory pathways resulted in better clinical outcomes at 6 months. Specifically, low disease activity measured by the Simplified Disease Activity Index (SDAI) and Disease Activity Score on 28 joints (DAS28) and ACR20 responses were achieved more often in the group of PsA patients receiving a tailored approach than in the conventional treatment approach group, in which no relevant biologic-dependent treatment decision was made. Similar outcomes were not obtained for cutaneous manifestations, for which the proportions of patients achieving PASI75 and PASI90 were not significantly different between the groups. According to the authors, it was the strategy itself that contributed to the achievement of a favorable response to treatment, instead of the type of bDMARD selected. This study, even if preliminary, is a forerunner in the application of a biomarker-driven approach to address conditions such as PsA.

#### Synovial Biomarkers

**FIGURE 1:**
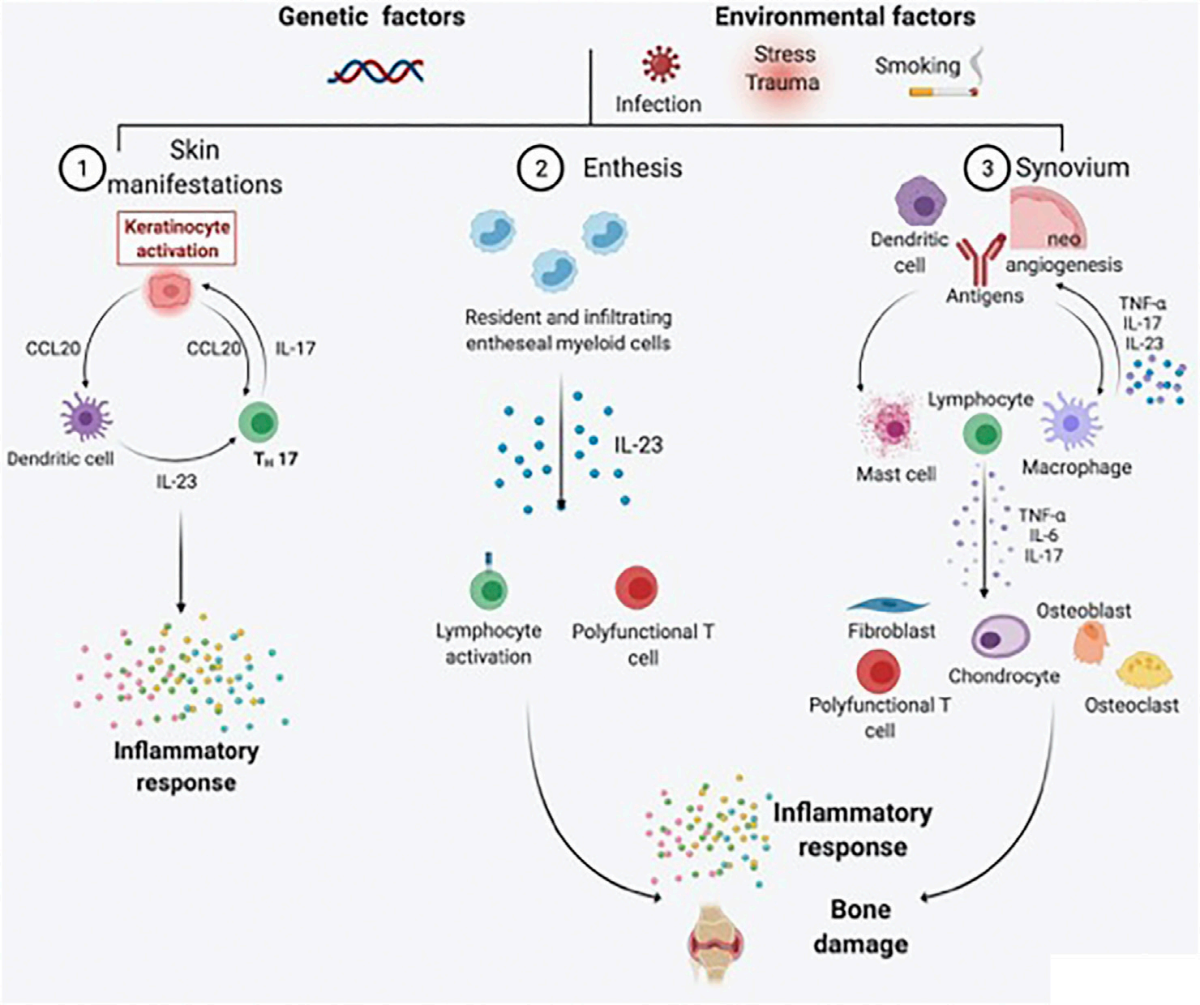
Schematic representation of the main pathogenic processes driving PsA development and chronicity. PsA is a heterogeneous chronic disease, with skin, entheseal, and synovial tissue involvement occurring in different proportions across patients under genetic and environmental triggers. For skin manifestations, activated dendritic cells secrete predominantly IL-23, which in turn induces the differentiation of naïve T cells into Th17 cells. IL-17 is responsible for keratinocyte activation and subsequent perpetuation of skin inflammation. At the entheseal level, resident and infiltrating entheseal myeloid cells produce IL-23, which is responsible for lymphocyte activation and the inflammatory response, as well as bone damage. Psoriatic synovitis, on the contrary, is characterized by tortuous and immature neoangiogenesis, with antigen presentation by dendritic cells and macrophages leading to lymphocyte activation. As a result, the synovial inflammatory infiltrate is rich in activated lymphocytes, mast cells, and macrophages. Polyfunctional T cells are responsible for the production of several types of proinflammatory cytokines (e.g., TNF-α, IL-17A, GM-CSF, and IFN-γ) in the synovial membrane and synovial fluid. The proinflammatory cytokine milieu further activates fibroblast-like synoviocytes, chondrocytes, osteoblasts, and osteoclasts, resulting in bone damage.

The analysis of cells and pathways in synovial tissue reveals findings that are not always exhibited by peripheral blood sampling. The anatomical proximity of the synovial membrane to the hypothesized inflammatory source emphasizes the putative roles of synovial biomarkers and their early modifications after treatment initiation. When chronic inflammatory arthritis occurs in the context of psoriatic disease, histological features include marked hyperplasia of the intimal lining layer containing fibroblast-like synoviocytes (FLSs) and macrophages and infiltration of the synovial sublining by both innate and adaptive immune cells, which are responsible for inflammatory mediator release, neoangiogenesis induction and cartilage and bone destruction. Inflammatory infiltrates in PsA consist of different immune cells, including macrophages, mast cells, polymorphonuclear (PMN) cells, and lymphocytes (B cells, T cells, and plasma cells), responsible for the significant production of different proinflammatory cytokines, including TNF, IL-1β, IL-6, IL-22, IL-23, IL-17A, and IL-18 (van Kuijk and Tak, 2011; Veale and Fearon, 2018) (Figure 1). Based on these observations, tracing inflammatory cells driving synovial inflammation in patients with undifferentiated inflammatory arthritis helped in the identification of tissue-dependent markers for predicting the development of defined chronic arthritis, such as PsA, within 1-year of follow-up (Alivernini et al., 2018). PsA synovitis partially differs from RA, as it is characterized by prominent neoangiogenesis with tortuous, immature, and elongated vessels (Kruithof et al., 2005a; Fromm et al., 2019); numerous macrophages in the lining (but not the sublining) layer (Rycke et al., 2005; Ambarus et al., 2012); and an increase in IL-17-positive infiltrating mast cells (Noordenbos et al., 2012). The presence of clonally expanded populations of CD8 T cells resistant to effective treatment (Curran et al., 2004; Penkava et al., 2020) suggests an antigen-driven T-cell response promoting inflammation. Although lymphoid aggregates and plasma cells are generally less represented in PsA than in RA (Alivernini et al., 2019; Nerviani et al., 2019), follicle-like structures found in the synovial tissue of treatment-naïve PsA patients are active, as shown by the presence of CD21^pos^ or CD23^pos^ follicular dendritic cells, together with the expression of activation/proliferation markers such as Ki67 and Bcl6, and are associated with the presence of autoantibodies in PsA patients at disease onset (Frasca et al., 2018). Moreover, consistent remodeling of bone metabolism is found in PsA, with bone neoformation markers interconnected with catabolic markers (allowing the presence of erosions along with new-bone growth) (Rahimi and Ritchlin, 2012; van Tok et al., 2018). The research utility of synovial biomarker discovery relies on the development of short-term clinical trials testing new drugs in early stages of pharmacological development (Gerlag and Tak, 2008; Codullo and McInnes, 2011), limiting the full period of the study to the time course of small proof-of-principle trials (“to-go-or-not-to-go”) (Sande et al., 2011). With this design in mind, studies investigating predictive synovial biomarkers of response to treatments have identified, in PsA, a reduction in sublining macrophages after effective TNFi treatment (Goedkoop et al., 2004a; Cañete et al., 2004; Rycke et al., 2005; Kruithof et al., 2006; van Kuijk et al., 2008; Pontifex et al., 2011). However, CD3^pos^ T-cell and MMP (MMP-3 and MMP-13) reductions appear to be the most sensitive biomarker variations associated with an effective treatment response to TNFis (Goedkoop et al., 2004a; van Kuijk et al., 2008; Pontifex et al., 2011; van Kuijk and Tak, 2011). Recent studies have used protein profiles generated from proteomic analysis of powdered synovial tissues to compare patients with a response to ETA and ADA therapy with nonresponders (Ademowo et al., 2016; Collins et al., 2016). Different sets of biomarkers have been proposed, involving acute-phase proteins, annexins, cytoskeletal proteins, the hypoxia response, angiogenesis, and apoptotic signaling. Again, validation of baseline synovial predictive biomarkers to demonstrate superiority for a biomarker-driven approach with respect to recommended treatment algorithms has not been undertaken to date.

On the contrary, studies investigating the synovial impact of drugs, mostly TNFis, have helped elucidate the effects of these drugs at the synovial level. TNFis, as an example, do not enhance apoptotic markers in either RA (Smeets et al., 2003) or PsA (Goedkoop et al., 2004a), but they are able to decrease inflammatory cytokine levels, interfere with inflammatory cell homing from the peripheral circulation via a reduction in chemokine and adhesion molecule production, and reduce neovascularization of the tissue (Baeten et al., 2001; Kruithof et al., 2005b; Gerlag and Tak, 2008). Researchers have found decreases in VEGF (Cañete et al., 2004), von Willebrand’s factor, αVβ integrin, and the adhesion molecules ICAM-1 and VCAM-1 (Baeten et al., 2001; Cañete et al., 2004; Goedkoop et al., 2004a, Goedkoop et al., 2004b) after TNFi treatment. Conversely, synovial mechanisms of the response to IL-17A blockers are not as widely understood. Van Mens et al. (2018) focused on longitudinal synovial modifications following SEC administration. After 12 weeks, there was a significant decrease in CD15^pos^ neutrophils and in CD68^pos^ macrophages in the sublining layer, with an increase in IL-17A-positive mast cells and reductions in IL-6, MMP-8, CCL-20, and IL-17A mRNA expression (Mens et al., 2018; Chen et al., 2019). The in vitro administration of an anti-IL-17A agent to FLS cultures was effective in reducing IL-17A-induced IL-6 production, with no differences between PsA and RA FLS (Frommer et al., 2019). SEC was also tested in vitro in SF mononuclear cell (SFMC) cultures and cocultures of FLS and peripheral blood mononuclear cells (PBMCs), producing a reduction in the release of monocyte chemoattractant protein 1 (MCP-1) after 48 hours (Nielsen et al., 2020). Finally, the adoption of a blocker of IL-17-receptor A (IL-17RA) was tested in PsA FLS cultures, highlighting reductions in IL-6 and IL-8 release into supernatants after stimulation with IL-17A (Raychaudhuri et al., 2012). In addition, the cellular effects of abatacept at the articular level (Szentpetery et al., 2017) have been longitudinally investigated in 14 patients starting abatacept or placebo treatment. Global synovitis and vascularity scores were significantly reduced after abatacept treatment compared with placebo treatment. The authors did not find significant changes in synovial CD3, CD8, or CD31 expression during the study period, while there was a significant reduction in FOXP3-positive CD4^pos^ regulatory T cells (Tregs).

Despite all these promising findings, these is not enough evidence to allow genetic, serum, cellular, or synovial biomarkers to be included in treatment decision-making in clinical practice, mainly because most studies were performed to assess only one class of drugs, without stratifying patient groups based on different treatment responses. Moreover, explanations for the effect of a single drug might be more complex than simply targeting a soluble molecule or receptor, given the heterogeneity of drugs in terms of structures and pharmacokinetics (Humby et al., 2017). Therefore, the main treatment selection rules remain mostly empirical, and treatment outcomes, both in RCTs and in clinical practice, remain essentially based on clinical measures.

### TNF-α-, IL-23/IL-17-, and JAK/STAT-Dependent Signal Transduction Axes

**FIGURE 2:**
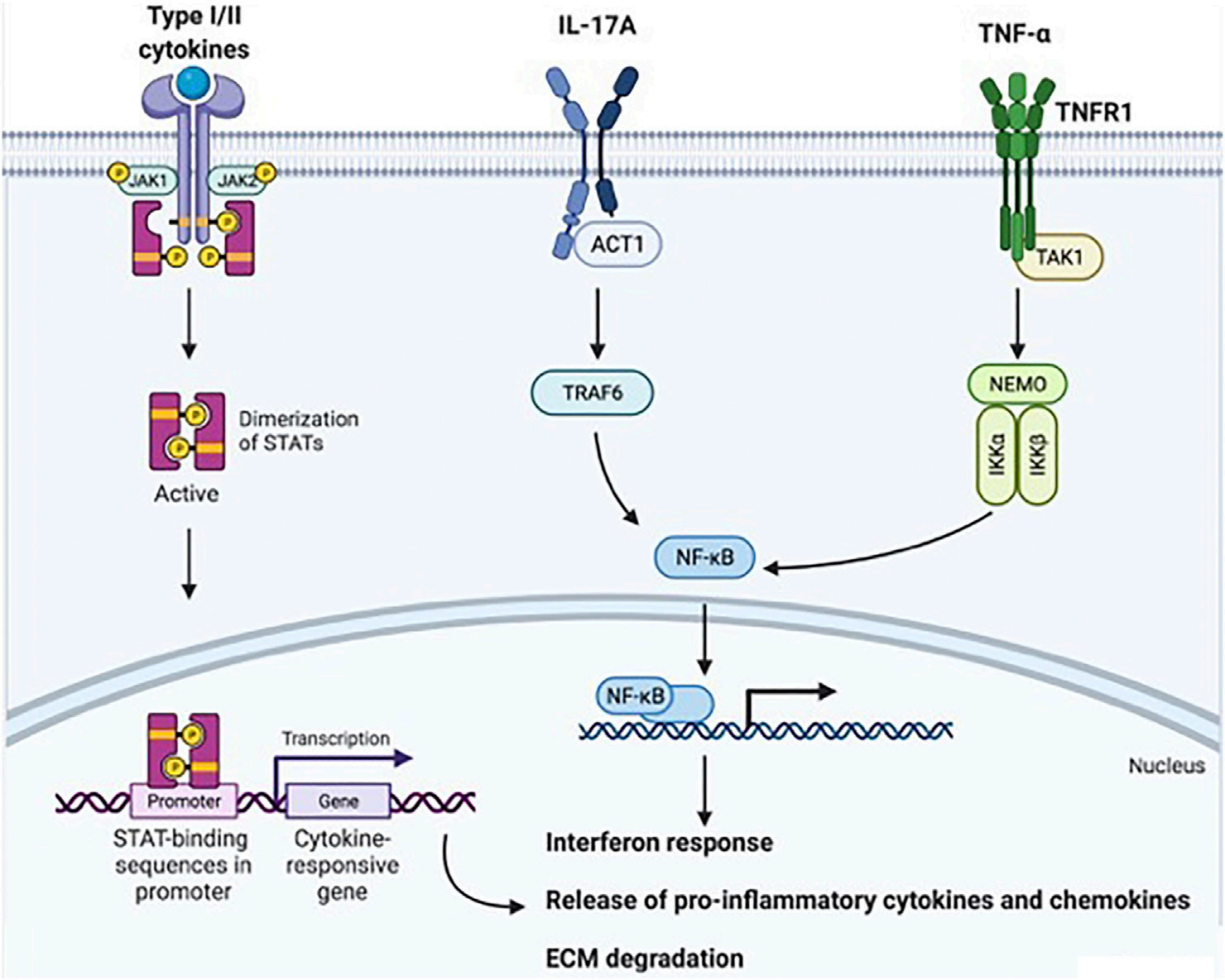
Downstream signal transduction mechanisms following TNF-α, IL-17A, or JAK/STAT-coupled receptor activation. Type I and II cytokines (e.g., IL-23, IL-22, IL-6, and type I, II, and III IFNs) bind JAK/STAT-associated receptors. JAK proteins are associated with the cytoplasmic domain of these receptors, and when cytokines bind to the receptor, JAKs undergo autophosphorylation and phosphorylate other JAKs. STATs recognize JAKs, bind their cognate receptors and become phosphorylated by JAKs. STATs then translocate to the nucleus, bind DNA and activate the transcription of target genes for the interferon response, proinflammatory mediator production, and ECM degradation. IL-17A binds IL-17R, a transmembrane heterodimer of IL17RA and IL-17RC. This binding induces Act-1 activation, which in turn activates TRAF6 and, accordingly, NF-kB. NF-kB migrates to the nucleus and induces target gene transcription. TNF-α, either in its soluble or transmembrane form, binds TNFR1 or TNFR2. After binding, in the classical proinflammatory axis induced by TNF-α-dependent cellular activation, TAK1 engages NEMO. NEMO activation results in the phosphorylation of specific serine residues in inhibitory proteins of NF-κB (IκBs) by IKK-1 and IKK-2, leading to IκB polyubiquitination and proteasome-dependent degradation. This process releases NF-κB proteins, which translocate to the nucleus and induce target gene transcription. Taken together, these mechanisms result in an interferon response, proinflammatory chemokine and cytokine release, and extracellular matrix degradation. Abbreviations: TNF-α, tumor necrosis factor-alpha; IL, interleukin; JAK/STAT, Janus Kinase/signal transducer and activator of transcription; ECM, extracellular matrix; IL-17R, IL-17 Receptor; Act1, NF-kappa-B activator 1; TRAF6, TNFR-associated factor 6; NF-kB, nuclear factor kappa-light-chain-enhancer of activated B cells; TNFR, tumor necrosis factor-alpha receptor; TAK1, transforming growth factor-alpha-activated kinase 1; NEMO, NF-κB essential modulator; IKK, inhibitor of IκB kinase; IκBs, inhibitory proteins of NF-κB. This image was created with © BioRender 2021.

Moving toward the bench side, the availability of efficacious drugs that directly target specific proinflammatory cytokines means, from a biological point of view, that interfering with TNF-α, IL-23/IL-17A axis, or JAK/STAT-dependent inflammation could disrupt several downstream signal transduction axes, with subsequent positive effects at the systemic and local levels. Knowledge of these signal transduction axes is, therefore, important to understand how each cytokine/node is dependent on or independent from others (Figure 2).

#### TNF-α

TNF-α is a key cytokine in the pathogenesis of SpA, and skin manifestations, as well as enthesis, joint, and spine involvement, represent the epiphenomenon of hyperactivated TNF-α-dependent inflammation as a result of innate and adaptive immune response activation (Palladino et al., 2003; Tracey et al., 2008; Croft and Siegel, 2017). TNF-α is part of the TNF superfamily, and its activities in health and pathology are pleiotropic. Many different immune and nonimmune cell types can produce this cytokine, including fibroblasts and keratinocytes. TNF-α can be found in either a soluble form or a transmembrane (tm) form bound to cells. The soluble form (sTNF-α) is released after enzymatic cleavage of the cell surface–bound precursor (tmTNF-α) by TNF-alpha-converting enzyme (TACE) (Higuchi and Aggarwal, 1994). Both sTNF-α and tmTNF-α are biologically active. TNF-α binds two distinct receptors: type I (TNFR1, also known as p55 and CD120a) and type II (TNFR2, also known as p75 and CD120b). Both receptors are transmembrane glycoproteins with multiple cysteine-rich repeats in the extracellular N-terminal domain. Signal transduction mediated by TNF-α receptor activation can alternatively lead to activation of nuclear factor kappa-B (NF-κB) or to apoptosis, depending on the metabolic state of the cell (Kuwano and Hara, 2000; Hayden and Ghosh, 2014). It is relevant to note that tmTNF-α can also act as a signal transducer and not only as a ligand. In this case, binding to tmTNF-α by TNFRs, or even TNFis, can induce reverse signaling and trigger cell activation or cytokine suppression and apoptosis in tmTNF-α-expressing cells. TNFR1 is constitutively expressed on virtually all nucleated cell types, whereas TNFR2 is inducible and preferentially expressed on endothelial and hematopoietic cells (Sedger and McDermott, 2014). The cytoplasmic region of TNFR1 contains a death domain that couples TNFR1 to either of 2 distinct signaling pathways via binding of the adapter protein TNFR-associated death domain. The first pathway leads to the activation of nuclear factor kappa-B1 (NF-κB1), a family of transcription factors that controls many inflammatory genes, while a distinct signaling pathway leads to caspase-8- and caspase-3-dependent apoptosis. In a “network concept” of the role of TNF-α in inflammation, TNF-α is considered an early and important trigger and mediator of downstream mechanisms, with a variety of feedback loops managing chronicity. However, it is not the only key cytokine involved in inflammatory pathways at the basis of chronic inflammatory arthritis development and perpetuation.

#### IL-17

IL-17-dependent signaling has been identified as a key modulator of synovial inflammation and joint destruction in various arthropathies, and its role in PsA pathogenesis, not only in skin manifestations (Robert and Miossec, 2019), is crucial (Doyle et al., 2012; Raychaudhuri et al., 2012; Raychaudhuri and Raychaudhuri, 2017). In particular, IL-17 is essential for increased expansion of Th17 cells, amplification and perpetuation of enthesitis, promotion of bone resorption via stimulation of receptor activator of nuclear factor-kB ligand (RANKL) expression, and modulation of inflammatory pain. The IL-17 family is composed of 6 different forms. IL-17A is the most active form, with 30-fold higher activity than IL-17F. IL-17A can also be part of an active heterodimer with IL-17F, which is thought to have intermediate activity between IL-17A and IL-17F. Cellular production of IL-17 is complex, and different cells are involved in its production. Naïve CD4^pos^ T cells that differentiate into Th17 cells in response to stimulation by IL-23 are considered the main producers of this cytokine, but other cell types (e.g., CD8^pos^ T cells, γδ T cells, NK cells, mast cells, polymorphonuclear cells, and group 3 innate lymphoid cells) consistently contribute to its production (Blijdorp et al., 2018; Chen et al., 2019). IL-17 receptor is a receptor complex formed by IL-17RA and IL-17RC (a heterodimeric transmembrane IL-17RA and IL-17RC complex). The binding of IL-17 to IL-17R leads to Act1 engagement, activation of the TNFR-associated factor (TRAF) 6 protein and subsequent NF-κB-mediated transcription of proinflammatory cytokines, among which IL-22 increases IL-17 function and activates osteoclasts and IL-21 promotes the differentiation of follicular Th cells. Conversely, IL-17E, also called IL-25, can bind another receptor formed by IL-17RA and IL-17RB, blocking downstream Th1/Th17 activation and, in contrast, increasing Th2 activity.

#### JAK/STAT-Dependent Signaling

In contrast, JAK/STAT-dependent signal transduction mediates the responses to a variety of different type I and II cytokines (Schwartz et al., 2016). It is relevant to note that neither TNF-α nor IL-17A signals via JAK/STAT-coupled receptors. However, IL-23, one of the key cytokines involved in Th17 polarization, and the IL-17-dependent downstream cytokines IL-22 and IL-21 bind to JAK/STAT-associated receptors. Type I cytokines include common gamma-chain cytokines (IL-2, IL-4, IL-7, IL-9, IL-15, and IL-21), common beta-chain cytokines (IL-3, IL-5, and granulocyte–macrophage colony-stimulating factor [GM-CSF]), IL-6, IL-23, and IL-12. Among type II cytokines, type I, II, and III IFNs and IL-10-related cytokines (IL-10, IL-22, and IL-20) are involved. JAK/STAT-coupled receptors have an extracellular cytokine-binding domain and a cytoplasmic domain that associates with JAKs. JAKs comprise four different proteins (JAK1, JAK2, JAK3, and tyrosine kinase 2 [TYK2]); when cytokines bind to the extracellular portion of their receptors, JAKs start working as phosphotransferases, transferring a phosphate group from ATP to tyrosine residues in their substrates. JAKs can transfer a phosphate group to themselves (autophosphorylation) or to other JAKs (transphosphorylation). Once JAKS are phosphorylated, they are recognized by STATs. The primary function of STATs relies on transmitting signals from type I and II cytokine receptors to the nucleus. There are currently seven known STATs: STAT1, STAT2, STAT3, STAT4, STAT5A, STAT5B, and STAT6. Prior to activation, STATs reside in the cytosol, but after cytokines bind to receptors, STATs bind to their cognate receptors and are phosphorylated by JAKs. After this modification, STATs translocate to the nucleus, where they bind DNA and activate the transcription of target genes, resulting in activation of key interferon response genes, production of proinflammatory cytokines and chemokines that perpetuate synovial inflammation, and activation of products that destroy the extracellular matrix (ECM) (e.g., MMPs), mediating cartilage and bone damage. Different JAK and STAT proteins can transmit signals from specific cytokines; however, a certain degree of functional promiscuity exists.

Among cytokines crucial in driving Th17 polarization, IL-23 is composed of two subunits, p19 and p40, which are linked by a disulfide bond. While the p19 subunit is an element unique to IL-23, the p40 subunit is shared with IL-12. IL-23 binds to its receptor IL-23R, which is coupled with JAK2 and Tyk2, members of the JAK family, which in turn mediate the downstream phosphorylation of STAT3 and its subsequent migration to the nucleus.

Given the broad range of cytokines that bind to JAK/STAT-associated receptors, targeting cytokines involved in JAK/STAT signal transduction could mean interfering with wider immune pathways than targeting only TNF-α or IL-17A per se, even if neither TNF-α nor IL-17A signaling occurs via JAK/STAT-coupled receptors. However, it is not clear how deep and complex the connections among these immune pathways might be.

### Relationships Among Different Proinflammatory “Immune Pathways” in PsA Synovitis

The clinical identification of patients refractory to specific targeting of TNF-α or the IL-23/IL-17 axis raises questions about the possibility of using a different available targeted therapy in refractory patients, with the aim of inhibiting the supposed “opposing” pathway. Few studies, however, have tried to investigate the specific clue regarding the connections between TNF-α and IL-23/IL-17 inflammatory effects in the context of PsA synovitis to clarify how targeting different nodes could modify their close interactions (Table 3).

**TABLE 3:** Main studies investigating the relationships between TNF-α- and IL-23/IL-17A-driven proinflammatory pathways in psoriatic synovitis.

Study	Type of study	Laboratory technique	Main results
Fiocco et al. (2010)	Longitudinal SF analysis before and at the last time SF sample was available for aspiration after IA-ETA treatment.	Luminex analysis	Longitudinal decreases in IL-1b, IL-6, and IL-22 levels in the SF AND no variation in IL-17A.
Noordenbos et al. (2012)	Longitudinal ST analysis before and after 12 weeks of systemic ETA treatment.	Double immunofluorescence	Longitudinal decrease in IL-positive cells AND no variation in IL-17-positive mast cells.
Mens et al. (2018)	Longitudinal ST analysis before and after 12 weeks of systemic SEC treatment.	Quantitative RT-PCR	Longitudinal reductions in IL-17A, IL-6, CCL-20, and MMP-3 mRNA levels AND no variations in IL-8 or TNF-α levels.
Chen et al. (2019)	Longitudinal ST analysis before and after 12 weeks of systemic SEC treatment.	IHC	Longitudinal reduction in IL-17A-positive non-mast cells AND increase in IL-17A-positive mast cells.
Raychaudhuri et al. (2012)	In vitro study, FLSs treated with IL-17A, TNF-α, and an IL-17RA blocker.	ELISA	TNF-α and IL-17A in vitro similarly increase IL-6, IL-8, and MMP-3 production in FLS cultures, while the IL-17RA blocker reduces the production.
Mitra et al. (2012)	In vitro study, FLSs treated with IL-22 and TNF-α.	Proliferation assays (MTT and CFSE dilution assays).	IL-22 and TNF-α-induced FLS proliferation AND IL-22 + TNF-α had a synergistic effect on FLS proliferation.
Gao et al. (2016)	In vitro study, synovial explant cultures treated with tofacitinib or DMSO.	ELISA	Ample effect of tofacitinib on in vitro cytokine production, reducing IL-8, IL-6, MCP-1, and MMP-3. IL-17A not detectable; TNF-α not evaluated.
Raychaudhuri et al. (2017)	In vitro study, SFMCs treated with tofacitinib.	FACS	Tofacitinib reduced IL-23-induced CD4 + CD11a + CD45RO + IL-17 + T cells.
Frommer et al. (2019)	In vitro study, FLSs treated with IL-17A, TNF-α, SEC, and ADA.	ELISA	IL-17A increases IL-6 release into FLS culture supernatants; TNF-α had a synergistic effect on increasing IL-6 release AND SEC and ADA had similar effects on IL-6 release inhibition.
Xu et al. (2020)	In vitro study, SFMC, and FLS coculture with SEC or ADA.	ELISA	SEC reduced IL-17A, IL-8 and IL-6 release; ADA reduced IL-8, TNF-α, and MMP-3/13.
Wade et al. (2019)	In vitro study, synovial cell suspensions treated in vitro with the PDE4 inhibitor rolipram.	ST single-cell suspension analysis + FACS.	Cells triple positive for GM-CSF, TNF-α, and IL-17 or IFN-γ were enriched in the PsA synovium compared to the peripheral blood and correlated with disease activity AND they were reduced by in vitro administration of rolipram.
Steel et al. (2020)	Cross-sectional study.	FACS	IL-17-positive CD8 T cells triple positive for GM-CSF, TNF-α, and IFN-γ, were enriched in the SF compared with the peripheral blood.

TNF-α, tumor necrosis factor-alpha; IL, interleukin; SF, synovial fluid; IA, intra-articular; ETA, etanercept; ST, synovial tissue; SEC, secukinumab; RT-PCR, real-time polymerase chain reaction; mRNA, messenger RNA; IHC, immunohistochemistry; FLS, fibroblast-like synoviocyte; IL-17RA, interleukin 17 receptor A; ELISA, enzyme-linked immunosorbent assay; MMP, matrix metalloproteinase; MTT, 3-(4,5-dimethylthiazol-2-yl)-2,5-diphenyltetrazolium bromide; CFSE, carboxy fluorescein succinimidyl ester; DMSO, dimethylsulfoxide; MCP-1, monocyte chemoattractant protein-1; SFMCs, synovial fluid mononuclear cells; FACS, Hi-D fluorescence-activated cell sorting; ADA, adalimumab; PDE4, phosphodiesterase-4; GM-CSF, granulocyte-macrophage colony-stimulating factor; IFN, interferon.

#### First Historical Highlights

In 2012, Noordenbos et al. (2012) investigated the role of mast cells in the pathogenesis of PsA synovitis. In their work, an analysis of paired synovial biopsy tissue samples obtained before and after 12 weeks of effective treatment with ETA demonstrated a significant decrease in the overall number of IL-17-positive cells, with no overall decrease in the number of IL-17-positive mast cells. The authors suggested that IL-17-positive mast cells could be resistant to clinically effective TNF-α inhibition. Similarly, Fiocco et al. (2010) demonstrated that PsA patients receiving intra-articular treatment with ETA showed reductions in the SF levels of different cytokines. As the main result of this study, post-treatment IL-1b, IL-6, and IL-22 levels were significantly reduced when compared with the corresponding baseline values, while IL-17A levels remained unchanged. In the same period, other researchers (Mitra et al., 2012; Raychaudhuri et al., 2012) investigated the effect of inhibiting the IL-17A axis in the PsA synovium. In a preliminary work, Raychaudhuri et al. (2012) cultured PsA FLS with IL-17A or TNF-α and found significant increases in IL-6 and IL-8 levels in both experiments. Blocking IL-17A activity with an IL-17RA blocker resulted in reductions in IL-6, IL-8, and MMP-3 production. In the work of Mitra et al. (2012), PsA-derived FLS cultured in the presence or absence of recombinant human IL-22, a proinflammatory cytokine that is produced by activated Th17 and Th22 cell subsets, induced FLS proliferation, an effect that was further enhanced when TNF-α was added to the culture. These studies have raised a relevant issue in the field of PsA concerning possible interference among multiple and different IL-mediated immune pathways using a “same targeted mechanism” drug. Conversely, they have cast doubts on the existence of different activated synovial proinflammatory immune processes in PsA patients, since a synergistic effect of IL-17A and TNF-α might be the most conceivable explanation.

#### Longitudinal Synovial Biopsy Studies

In fact, the recent availability of anti-IL17A agents for the clinical management of PsA provides an opportunity to explore the synovial effects of SEC in terms of interactions with TNF-dependent inflammation. In a recent report by Van Mens et al. (2018), the authors performed synovial biopsies before administering the anti-IL17A SEC to peripheral SpA patients, including PsA subjects, and found significant reductions in IL-17A, IL-6, and MMP-3 mRNA levels but not in TNF-α levels 12 weeks after the start of the new drug. Furthermore, Chen et al. (2019) demonstrated that the number of IL-17A-positive mast cells was increased after 12 weeks of SEC treatment in peripheral SpA patients (particularly when a clinical response was achieved), while that of IL-17A-positive non-mast cells was significantly reduced, suggesting different types of IL-17 metabolism in different types of immune cells.

#### In Vitro Culture Systems

Exploiting in vitro culture systems, other researchers have tried to solve the problem from another perspective. Frommer et al. (2019) demonstrated a synergistic effect for IL-17A and TNF-α in promoting IL-6 release into culture supernatants of PsA-FLS, while ADA and SEC were similarly effective in inhibiting IL-6 release. In 2016, another group of researchers (Gao et al., 2016; McGarry et al., 2017) performed studies investigating the synovial effect of the tsDMARD tofacitinib in PsA. In their works, the authors cultured PsA-derived FLS and whole-tissue synovial explants in the presence or absence of 1 µM tofacitinib citrate. Tofacitinib, deemed to have a wider synovial effect than TNF-α inhibitors and IL17A blockers, inhibited PsA-derived FLS invasion and migration and reduced IL-6, IL-8, and monocyte chemoattractant protein-1 (MCP-1) release into supernatants compared with a vehicle control. In this work, however, the IL-17A levels in PsA synovial explant cultures were undetectable, while TNF-α levels were not investigated. Raychaudhuri et al. (2017) demonstrated that tofacitinib was able to reduce the number of IL-23-induced IL-17-positive memory T cells in PBMCs and SFMCs derived from PsA patients. As it is known that neither IL-17A nor TNF-α signals via the JAK/STAT pathway, one of the explanations for the link between tofacitinib treatment and IL-17-positive T-cell reduction is the inhibition of IL-23 activity (which signals via JAK/STAT-coupled receptors) by tofacitinib and the associated further reduction in IL-17A production by different downstream effectors. This interconnection among several cytokines remains crucial when considering the complex relationships among different inflammatory pathways. In fact, it has been demonstrated in RA that TNF-α and the IL-23/IL-17A axis produce synergistic effects on bone damage (Kirkham et al., 2006) and chemotactic activity toward T cells and dendritic cells in the synovium (Chabaud et al., 2001). One of the mechanisms thought to mediate IL-17A and TNF-α synergism involves inflammatory protein mRNA transcript stabilization, independent of TRAF-6-dependent signal transduction (Hartupee et al., 2009). The synergistic effect of IL-17 and TNF-α might be dependent on the cell type, as demonstrated in several cell cultures, including RA-FLS (Noack et al., 2019) and CD14pos myeloid cell populations in normal enthesis soft tissue and perientheseal bone (Bridgewood et al., 2019). Moreover, since several cellular elements are key characteristics of psoriatic synovitis, the adoption of more sophisticated in vitro models of chronic synovitis might be of value. Xu et al. (2020) co-cultured CD4 T cells isolated from PsA SF with allogeneic FLS and treated the in vitro system with ADA or SEC. SEC significantly reduced IL-17A and IL-6 release into the cellular supernatant, while ADA reduced TNF-α and MMP-3/13. Both drugs reduced IL-8 levels, while IFN-γ was not reduced by either of the treatments. In summary, although SEC and ADA showed overlapping activity affecting mediator release, a differential effect was demonstrated, with the anti-IL-17A agent mostly inhibiting inflammatory mediator release and TNF-α antagonism impacting structural mediators (e.g., MMPs).

#### Lessons from Genetics, Animal Models, Skin, and Systemic Counterparts

As it is not completely accepted that the synovium is the appropriate tissue to evaluate the etiopathogenesis of psoriatic disease, in which skin and entheseal manifestations account for a large part (Figure 1), and given the relative difficulty of obtaining samples of entheseal or bone tissue from live patients, researchers have tried to investigate the specific clue about the presence of different/opposing pathways in PsA using animal models of arthritis, as well as cutaneous biopsies, circulating cells, and genomic profiling studies. Belasco et al. (2015) analyzed paired PsA synovial tissue and skin samples. They demonstrated higher IL-23/IL-17-related gene expression in cutaneous biopsies, while TNF and IFN-γ-signatures were quite homogenously expressed in both sites. These findings point toward the existence of distinct phenotypic inflammatory activities that govern pathology in the skin compared with that in the joints but do not explain why a significant proportion of patients with polyarthritis become refractory to TNFis. Recently, these data were confirmed by Nerviani et al. (2020), who evaluated 14 matched synovial tissue and skin biopsies from PsA patients. The relative gene expression of TNF-α was homogeneous in both the skin and the synovium, while IL-23A, IL-12B, and IL-23R showed higher expression in lesional skin than in the synovium.

Studies of murine models of arthritis are helpful in understanding links between TNF- and IL-17-dependent inflammation and have shown that treating collagen-induced arthritis (CIA) mice with TNF-blocking agents results in a rebound increase in lymph node Th17 cells, with a converse reduction in synovial Th17 cells (Notley et al., 2008). Similar rebounds in splenic and lymph node Th17 cells were also demonstrated in genetic TNFR2 loss-of-function mice (Miller et al., 2015). The addition of IL-17A was able to induce an experimental model of arthritis independent of TNF (Koenders et al., 2005b; Koenders et al., 2005a, Koenders et al., 2006). Studies of murine models of psoriasis have similarly proven increases in cutaneous and serum IL-17A and IL-22 levels after TNF-α blockade (Ma et al., 2010). Whether these findings apply to patients with exacerbated skin psoriasis under TNFi treatment (Brown et al., 2017) is not known. The results obtained in mouse models of arthritis show promise for clarifying the relationships among different immune pathways but—at the same time—are challenging the assumption that these two cytokine subsets are mutually antagonistic. Again, these mouse models are not fully generalizable to humans, indicating the need for further research.

In fact, studies of human patients with skin psoriasis have failed to demonstrate rebounds in IL-17A and IL-22 at the cutaneous level after TNFi treatment (Zaba et al., 2007, Zaba et al., 2009; Johnston et al., 2014), despite an increase in circulating Th17 cells (Hull et al., 2015). Tissue resistance to IL-17A, mediated by downregulation of IL-17RC, was found to be an early modification after ETA treatment in psoriatic patients. Cutaneous expression of IL-17A-related genes was unchanged in cutaneous biopsies from refractory patients (Zaba et al., 2009). Similar results were obtained following ineffective tofacitinib treatment (Krueger et al., 2016). With particular regard to blood biomarkers, Kim et al. (2018) investigated the effect of systemic treatment with etanercept or tofacitinib in psoriatic patients on relevant proinflammatory and cardiovascular protein biomarkers. The main result of their work was that after 4 weeks of treatment, both tofacitinib and etanercept reduced IL-6, CCL20, and CXCL10 levels, but the IL-17A level was significantly reduced only in responders for either treatment. Recently, in a study by Miyagawa et al. (2018), systemic treatment with SEC resulted in a reduction in circulating Th17 cells and an increase in Th1 cells. The authors’ explanation of this rebound relied on the inhibition of the expression of IL-12 receptors on naïve T cells modulated by IL-17. The reduction in IL-17 levels after SEC treatment could revert this inhibitory effect, thus facilitating the expression of IL-12 receptors and inducing differentiation into Th1 cells. Nonetheless, this rebound was not associated with clinical worsening of disease.

#### Future Perspectives for Precision Medicine Approaches

Considering the synovial environment, Wade and coworkers (Wade et al., 2019; Canavan et al., 2020) investigated the presence of polyfunctional T cells in synovial tissues from PsA patients, hypothesizing that an effective treatment could interfere with this specific cluster of cells, which are able to produce a wide variety of different cytokines. The authors demonstrated that a significant proportion of synovial T-cell subsets were triple-positive for GM-CSF, TNF, and IL-17 or IFN-γ compared with matched blood subsets and that these polyfunctional T cells were positively correlated with disease activity (measured with the Disease Activity in PSoriatic Arthritis (DAPSA) score), while single cytokine-producing T cells were not. Moreover, in vitro administration of a phosphodiesterase 4 (PDE4) inhibitor (rolipram) to synovial cell cultures significantly reduced the number of polyfunctional triple-positive T cells. Additionally, CD8 T cells were found to be polyfunctional in PsA SF, producing IL-17A, IFNγ, TNF-α, GM-CSF, and IL-22 (Steel et al., 2020). These studies suggest that a single T-cell population might be able to orchestrate diverse inflammatory pathways, specifically at the synovial level, and treatments able to interfere with the activity of all of these pathways, or strategies based on the recognition of patterns of cytokine expression at the single-patient level, might be more effective than others targeting a single mechanism or determined empirically.

The possibility of translating this seminal evidence into clinical practice is fascinating. In a similar condition such as RA, a recent RCT, for the first time, exploited information derived from synovial histology and mRNA expression analysis to inform selected treatment decisions (Humby et al., 2021). The combination of peripheral T-cell phenotyping (Miyagawa et al., 2018) with single-cell analysis at target tissue levels might also be used to inform treatment schedules in the context of PsA. In particular, not only cutaneous samples but also the synovial membrane should be evaluated to identify, at a particular time point in the disease history, the most active pathway at the single-patient level. In addition, it is still not known how “big data” analytics (e.g., machine learning and artificial intelligence) might impact personalized medicine approaches, providing individual molecular and clinical data to be compared with population-based data (Ritchlin et al., 2020b). Since rapid evolution is expected in the near future, the possibility of obtaining data from international multicenter consortia involving different types of target tissues, along with a refined definition of clinical endotypes linked with drug response or sequential treatment history, might impact management schedules and timelines, definitely changing the “heuristic” strategy to a personalized “precision medicine” approach.

## Discussion and Conclusion

The rules driving the treatment paradigm of “the right drug for the right patient at the right moment” in PsA have not been determined yet. At the bedside, many treatments with different mechanisms of action have been proven to be effective, and from a clinical point of view, it seems reasonable to think that TNF-α and IL-23/IL-17A inhibitors block different pathogenic processes, with JAKis inhibiting a wider number of cytokine nodes. However, it is unclear whether the clinical response to TNFis implicitly indicates a TNF-α-driven disease not amenable to IL-23/IL-17 targeting. Since biomarkers have not entered clinical practice to drive treatment decisions in PsA, the paradigms guiding therapy selection, and strategy adoption remain almost empirical. In contrast, at the bench, the analysis of cytokine downstream signaling pathways has revealed several axes that only appeared to be disconnected, and studies analyzing longitudinal synovial tissue modifications after the start of TNFis or IL-17A blockers, as well as in vitro models of PsA synovitis, genomic profiling studies, animal models, and skin or peripheral blood cell population analyses, have revealed much more complex interconnections. TNF-α and IL-17A demonstrate overlapping and synergistic activities, with differential variations depending on the type of cells analyzed, creating the possibility of “adaptation” to alternative key nodes at the tissue level. Again, the presence of polyfunctional cells pertaining contemporarily to alternative nodes might be central in driving disease aggressiveness and treatment resistance. Therefore, the analysis of tissue-derived samples might help in unveiling the interconnections among different nodes and selecting the most active node (or the driving pathway) suitable for targeting by available drugs to optimize an even more ambitious approach based on “precision medicine” in this highly challenging chronic disease, in accordance with a “patient-centered” perspective.
